# Proteinase-Activated Receptor 2 (PAR2) Deficiency and Cardiovascular Regulation: Context-Dependent Effects on Inflammation and Fibrosis

**DOI:** 10.3390/cimb48080821

**Published:** 2026-08-12

**Authors:** Stephanie A. Viola, Shahnaz Siddiqua, Jesutofunmi Adesuyi, Yebin Jang, Maryia Ryskina, John J. McGuire

**Affiliations:** 1 Department of Medical Biophysics, Schulich School of Medicine & Dentistry, Western University, London, ON N6A 5C1, Canada; sviola3@uwo.ca (S.A.V.); ssidd3@uwo.ca (S.S.); jadesuyi@uwo.ca (J.A.); mryskina@uwo.ca (M.R.); 2 Department of Physiology and Pharmacology, Schulich School of Medicine & Dentistry, Western University, London, ON N6A 5C1, Canada

**Keywords:** proteinase-activated receptor 2 (PAR2), cardiovascular regulation, vascular inflammation, cardiac fibrosis, context-dependent signalling

## Abstract

Proteinase-activated receptor 2 is a G protein-coupled receptor that regulates vascular tone and inflammatory signalling in the circulatory system. The roles of PAR2 appear complex and sometimes opposing. Studies using PAR2-deficient mice provide a framework to define these effects at the system level. This review examines cardiovascular phenotypes associated with PAR2 deficiency in basal conditions and in disease. PAR2 deficiency produces modest increases in arterial blood pressure and vascular stiffness while preserving endothelial vasodilator function. Cardiac function remains largely normal in young PAR2-deficient animals but changes with age. Older PAR2-deficient mice develop diastolic dysfunction and cardiac fibrosis. In disease models, PAR2 deficiency has been associated with increased fibrosis in cardiac and vascular tissues and reduced vascular inflammation in atherosclerosis. PAR2 deficiency is also associated with reduced plaque progression and features of plaque stabilisation. In myocardial ischaemia models, PAR2 deficiency has been associated with reduced cardiac injury and adverse remodelling. The effects of PAR2 deficiency on inflammatory signalling vary according to tissue and disease context. Together, these findings suggest that the cardiovascular consequences of PAR2 deficiency depend on physiological and pathological context. Future studies should define cell-specific mechanisms to guide therapeutic targeting of PAR2.

## 1. Introduction

Proteinase-activated receptor 2 (PAR2) signalling plays a crucial role in blood vessel functions and affects the circulatory system in health and disease. PAR2 is a product of the Factor 2 (thrombin) receptor-like 1 gene, *F2RL1* and *f2rl1*, in humans and mice. Decades of studies using *f2rl1*-deficient (PAR2^−/−^) mice [1,2] have demonstrated cardiovascular phenotype differences in arterial blood pressures [3], cardiac function with ageing [4], and both cardiac and vascular tissue fibrosis [4,5,6]. PAR2^−/−^ mice also exhibit differences in cardiovascular recovery from myocardial infarct [7,8,9] and cardiac ischaemia-reperfusion injury [10], and altered responses to Coxsackievirus B3 virus-induced myocarditis [11], blood vessel inflammation, e.g., atherosclerosis [12,13,14], and trauma [15], vascular smooth muscle phenotype transition [16], acute and chronic kidney injuries [17,18,19], and retinal and hindlimb ischemia [20,21,22]. However, PAR2^−/−^ mice have not only a decrease in PAR2 signalling but also an increase in PAR1 [4,23,24]. Altered PAR1/PAR2 signalling has also been reported in patients with heart failure with preserved ejection fraction (HFpEF) [4]. Whether these clinical observations reflect mechanisms similar to those observed in PAR2^−/−^ mice remains uncertain. The specific contributions of vascular endothelium, vascular smooth muscle, and cardiomyocytes, which express PAR2 in both mice [25] and humans [26], to the PAR2^−/−^ phenotype remain largely undetermined. The unique molecular mechanisms underlying the pathophysiological activations of PAR1 and PAR2 provide an experimental framework for discovering novel pharmacological modulators. This framework, aided by PAR2^−/−^ mice, helps explore the function of PAR2 in the circulatory system. Furthermore, the tethered ligand activation mechanism suggests PAR-subtype-specific pharmacology and highlights PAR2 as a transducer of intercellular signalling.

PAR2 is a G protein-coupled receptor (GPCR) and one of four in the PAR family [27]. Extracellular proteases such as trypsin, trypsin-like serine proteases, and human mast cell β-tryptase activate PAR2 via a tethered ligand mechanism that is unique to the PAR family [28]. Proteolytic cleavages of the extracellular N-termini of PAR1 and PAR2 reveal tethered ligands that bind to the receptors to induce conformational changes leading to GPCR signalling [29,30]. The structure-activity relationships of the tethered ligands for each PAR and the protease-selectivity for recognition of the N-terminus as a substrate hold a long-standing interest in academic and pharmaceutical industry research but are beyond the scope of this review. Readers are directed to [31] for a comprehensive, consolidated online pharmacological database on the PAR family. Synthetic peptide agonists referred to as PAR2-activating peptides (PAR2-AP) mimic the tethered ligand sequence and directly activate PAR2 in the absence of proteolysis. PAR2-APs are key tools used to develop small-molecule PAR2-modulating compounds, i.e., both agonists and antagonists. PAR2-APs, such as the amidated forms of Ser-Leu-Ile-Gly-Arg-Leu (SLIGRL) and 2-furoyl-Leu-Ile-Gly-Arg-Leu-Orn (2fLIGRLO), were tested in studies with PAR2^−/−^ mice to validate specificity [32,33]. The use of PAR2^−/−^ mice has contributed to the rationale for pursuing PAR2 inhibitors and receptor antagonists, particularly in chronic inflammation [34]. Understanding PAR2 activation and signalling is foundational to investigating its diverse roles and advancing PAR2 therapeutic discovery.

The vascular endothelium is a target of constitutive PAR2 signal transduction within the circulatory system. PAR2 activation decreases vascular tone by endothelium-dependent mechanisms specific to vessel type [32,33]. PAR2-AP administered in vivo lowered blood pressure in rodents [2,23,35,36] and increased forearm [37,38] and cutaneous [39] blood flows in humans. However, treatment of human endothelial cells with PAR2 activators increased surface expression of P-selectin and neutrophil adhesion, induced expression of cyclooxygenases, and increased cytokine release [40,41]. Pro-inflammatory cytokines such as tumour necrosis factor-α (TNF-α) and interleukin-1α (IL-1α) induce PAR2 expression in human endothelial cells and blood vessels [42,43]. Thus, in endothelial cell systems, PAR2 activation may enhance inflammatory responses by increasing permeability and adhesion molecules or can preserve vasodilation/relaxation, depending on the mediator balance (nitric oxide (NO) vs. cytokines). Therefore, PAR2 can appear “protective” in some vascular beds but deleterious in others, without contradiction. Where constitutive expression of PAR2 in other cardiovascular cell types, i.e., cardiomyocytes, myofibroblasts, and vascular smooth muscle, is less evident than in vascular endothelial cells of adult mice [25], the functions of PAR2 have been examined mostly in the context of modifying disease and recovery from injury.

The predominant perspective from most reviews has been that PAR2 primarily mediates pro-inflammatory pathways in diseases and tissue injury, as evidenced by studies showing its involvement in conditions like atherosclerosis and myocarditis. Initial in vivo studies with PAR2^−/−^ mice found evidence of a protective effect of PAR2^−/−^ against acute inflammation of lung airway epithelium [1]. Since then, research studies have evaluated PAR2^−/−^ in chronic inflammation and related nociception pathways, which encompass many different organs, tissues, and cell types. Nevertheless, countervailing evidence obtained using PAR2^−/−^ mice has shown a role for PAR2 in anti-inflammatory activities and promotion of tissue repair mechanisms. The differences in these perspectives drawn from PAR2^−/−^ mice appear to align with the nature of the inflammation and injury models and the individual responses of specific tissues and organs. This review focuses on studies using PAR2^−/−^ mice to advance understanding of intercellular signalling in the cardiovascular system and highlight the context-dependent roles of PAR2.

## 2. PAR2-Deficient (PAR2^−/−^) Mice

### 2.1. Hemodynamics and Cardiac Performance

PAR2^−/−^ mice exhibit modest differences in cardiovascular physiological characteristics, including approximately 3–4% (4–5 mm Hg) higher systolic arterial blood pressure and 6–10% (2–3 mm Hg) higher pulse pressure compared with wild-type (WT) mice of the corresponding genetic background strain [3]. Earlier studies in anaesthetised animals reported no differences in the baseline mean arterial pressures between PAR2^−/−^ and WT mice [23], which could be attributed to methodological differences in blood pressure measurements. Studies have examined whether the small changes in blood pressures may result from increased peripheral resistance caused by higher sympathetic-driven basal tone or a loss of endothelium-derived mediators of vasodilation in PAR2^−/−^. Ex vivo assays using resistance arteries indicated a higher sensitivity to α1-adrenoceptor vasoconstrictor agonists in PAR2^−/−^ compared to WT mice [44]. However, in blood vessels from PAR2^−/−^, no reductions of either endothelium-dependent or -independent relaxation mechanisms have been reported [24,32,44,45]. We have speculated that the reported small increases in arterial pulse pressure in PAR2^−/−^ resulted from lower systemic vascular compliance, caused by stiffening of vasculature due to morphological or structural changes such as fibrosis of blood vessel walls, and an increase in cardiac stroke volume in PAR2^−/−^. Indeed, studies have found elevated pro-fibrotic pathway activity in systemic conductance arteries, i.e., thoracic aorta, and cardiac left ventricles of PAR2^−/−^ [4,5,6]. Thus, in PAR2^−/−^ mice, a phenotype has emerged indicating small increases in blood pressure and a propensity for developing vascular and cardiac fibrosis.

Under conditions such as a normal diet and absence of stressors, PAR2^−/−^ does not significantly impact renal blood pressure regulation. In PAR2^−/−^, the blood pressure-raising effects of a high-salt diet and chronic infusion with high angiotensin II did not differ from those in WT at the start and end of treatments [3]. Similarly, under basal, salt-loading, or agonist-stimulated conditions, plasma renin activity did not differ between PAR2^−/−^ and WT [46]. However, PAR2 deficiency modified renin responses in inflammatory states [46] as well as glomeruli inflammation and fibrosis pathways in both acute [17] and chronic [18,19] models of kidney disease. The effects of PAR2 deficiency on renal inflammation are still context-dependent given evidence that PAR2^−/−^ did not differ from WT in a ureteric obstruction model of renal tubular injury [47]. Thus, at least in some kidney disease models with inflammation components, which included normal ageing, PAR2^−/−^ may be expected to impact renal regulation of blood volume and pressure via changes to glomerular filtration and natriuresis.

**Figure 1:**
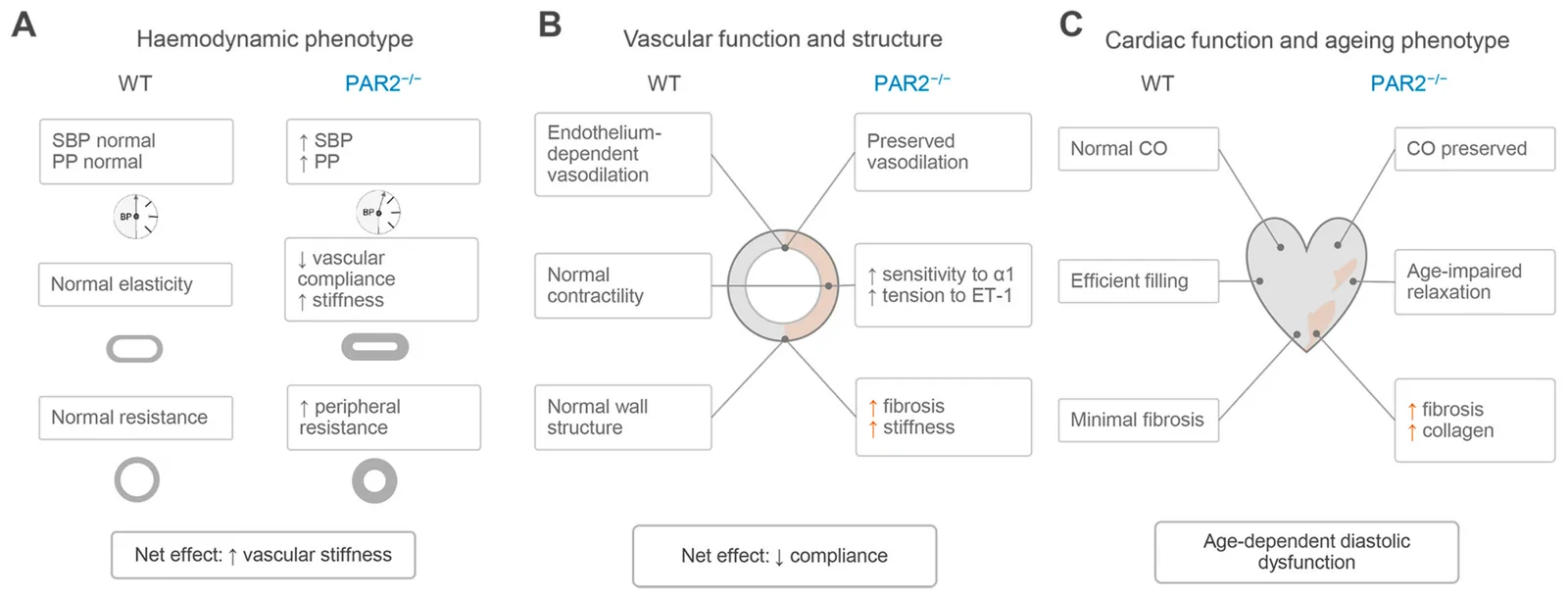
Hemodynamic, vascular, and cardiac phenotypes associated with PAR2 deficiency. (**A**) PAR2^−/−^ is associated with modest increases in systolic blood pressure (SBP) and pulse pressure (PP), reduced vascular compliance, and increased peripheral resistance, consistent with increased vascular stiffness. (**B**) Vascular responses show preserved endothelium-dependent vasodilation alongside enhanced vasoconstrictor sensitivity and structural remodelling, including increased fibrosis and stiffness. (**C**) Cardiac function is maintained at the systolic level, while diastolic relaxation is impaired with age and accompanied by increased fibrosis and collagen deposition. Together, these findings indicate that PAR2 deficiency produces modest hemodynamic changes but promotes vascular stiffening and age-dependent cardiac dysfunction. This figure is an original conceptual summary created by the authors to synthesize cardiovascular phenotypes associated with PAR2 deficiency reported in the reviewed literature. Abbreviations: α1-adrenoceptor; CO, cardiac output; ET-1, endothelin-1; PAR2, proteinase-activated receptor-2; PP, pulse pressure; SBP, systolic blood pressure.

Studies show cardiac function changes in PAR2^−/−^ mice over their lifespan. In young adults (12 weeks of age), cardiac stroke volumes, output, ejection fractions, and ventricular pressure rates did not differ from WT mice [4,11]. However, diastolic dysfunction and cardiac fibrosis were found in aged (1-year-old) PAR2^−/−^ mice [4]. Pressure-volume microcatheter measurements of these aged PAR2^−/−^ mice indicated that systolic cardiac performance and efficiency (ejection fraction; systolic ventricular pressure rate change, end-systolic pressure) did not differ from controls, but diastolic functions (left ventricular relaxation rates and time-constants, end-diastolic pressures) in PAR2^−/−^ differed from 1-year-old WT mice. Further, the study reported that the hearts of aged PAR2^−/−^ mice exhibited higher PAR1 activity, which was linked to suppressed caveolin-1 regulation of PAR1 via vesicle trafficking and expression on the plasma membrane [4]. PAR1 upregulation has been reported in several studies using PAR2^−/−^ mice [23,24,32]. Friebel et al. [4] linked PAR2^−/−^ cardiac myofibroblast expression of PAR1 to transforming growth factor-β (TGF-β) as a stimulus for collagen production, leading to cardiac fibrosis. Deposition of collagen in the myocardium is associated with stiffening of the left ventricle, which is assessed by the parameters of cardiac diastolic function. Direct investigation of the relationship between the PAR2^−/−^ blood pressure phenotype and cardiac diastolic dysfunction has not been made. Further, the occurrence of diastolic dysfunction was associated with normal cardiac output in PAR2^−/−^. In animal models of metabolic syndrome, interactions between age and diminished PAR2 have been described with respect to endothelial dysfunction in blood vessels [48]. Thus, PAR2^−/−^ may confer additional risks to circulatory dysfunction associated with ageing and metabolic diseases. These findings define a system-level cardiovascular phenotype associated with PAR2 deficiency (Figure 1).

### 2.2. Circulatory System Under Stress

Studies using PAR2^−/−^ in models of cardiac and circulatory dysfunction provide evidence of a role for PAR2 in mechanisms of disease and injury, particularly linked to fibrosis. At 8 months of age, PAR2^−/−^ mice were found to have higher endothelin-1 (ET-1) plasma levels than WT mice, and PAR2^−/−^ mouse aortas ex vivo produced more contractile tension in response to ET-1 [5]. Elevated circulating levels of vasoconstrictor molecules such as endothelin-1 can lead to vascular fibrosis in mice. Interestingly, in tight-skin mice, a preclinical model of scleroderma-like syndrome characterised by blood vessel fibrosis and contractile dysfunction, the responses of aortas to PAR2 activation were enhanced [5]. In a model of angiotensin II-induced hypertension with cardiac myocardium remodelling, PAR2^−/−^ was associated with enhanced cardiac interstitial fibrosis, which was interpreted as evidence suggesting a potential anti-fibrotic role for PAR2 [6]. The role of PAR1 upregulation in the pro-fibrotic effects of PAR2^−/−^ in these vascular models remains unclear.

Across independent hyperlipidaemic mouse models, PAR2 deficiency has been associated with reduced atherosclerotic lesion progression, lower inflammatory mediator expression, and features of plaque stabilisation. These observations suggest that PAR2 may contribute to inflammatory signalling pathways involved in atherosclerosis. In the context of atherosclerosis, available evidence suggests that PAR2 signalling may promote inflammatory responses associated with plaque progression. In experiments using PAR2^−/−^ bred with atherosclerosis-prone mice strains, i.e., apolipoprotein E gene-deficient (*apoE*^−/−^) and low density lipoprotein receptor gene-deficient (*ldlr*^−/−^), plaque lesion area and macrophage accumulation in mice fed a high-fat diet for 8 weeks were less in the double gene knockouts, i.e., *apoE*^−/−^/PAR2^−/−^, and *ldlr*^−/−^/PAR2^−/−^, than in age-matched single gene knockouts [12,13,14]. Lower macrophage accumulation, higher α-smooth muscle actin (α-SMA) expression, thicker fibrous caps, and higher collagen content in plaques were interpreted as evidence that *apoE*^−/−^/PAR2^−/−^, *ldlr*^−/−^/PAR2^−/−^ exhibit a more stable plaque phenotype [12,14]. Pro-inflammatory mediators, monocyte chemoattractant protein-1 (MCP-1), tumour necrosis factor-α (TNF-α), interleukin-6 (IL-6), matrix metalloproteinase-9 (MMP-9), and C-X-C motif chemokine ligand 1 (CXCL1) expressions were less in *apoE*^−/−^/PAR2^−/−^ than *apoE*^−/−^ [12,13,14]. Indeed, inhibitor of nuclear factor-kappa B protein-alpha (IκBα), a tissue biomarker of activity known to correlate with lower activation of the pro-inflammatory nuclear factor-kappa B (NF-κB) signalling pathway, was higher in *apoE*^−/−^/PAR2^−/−^ than *apoE*^−/−^ [14]. Importantly, across studies, the PAR2^−/−^ effects were attributed to local vascular pathology, not systemic lipid metabolism or blood pressures. Bone marrow transplantation experiments using the PAR2^−/−^ provided evidence that monocyte PAR2 expression drove the vascular inflammation in these models [13,14]. Nevertheless, in vitro studies with the PAR2^−/−^ cells suggest the potential for an autocrine signalling between macrophages and vascular smooth muscle that could modulate the net or temporal response [13]. Cultured VSMCs lacking PAR2 produced less MCP-1 and CXCL1, suggesting PAR2 may contribute to monocyte recruitment from a non-haematopoietic source. These findings suggest that PAR2 may amplify inflammatory responses across multiple vascular compartments rather than acting exclusively through a single cell type. Thus, in atherosclerosis, studies of PAR2^−/−^ mice have identified PAR2 as a potential therapeutic target.

PAR2 modulates injury-induced blood vessel remodelling and associated vascular disease progression. PAR2^−/−^ decreased neointimal hyperplasia, inflammatory cell infiltration, and smooth muscle cell accumulation following vascular injury. Extending this work into true aneurysm pathology, PAR2^−/−^ attenuated abdominal aortic aneurysm progression in mice by preserving contractile VSMC phenotype and limiting ERK-dependent proliferation and migration, implicating PAR2-mediated smooth muscle plasticity as a driver of aneurysmal remodelling [16]. Thus, from a clinical translation perspective, research with PAR2^−/−^ has built a case for PAR2 as a therapeutic target for slowing abdominal aortic aneurysm progression, not necessarily preventing onset.

Circulatory collapse and multi-organ failure leading to death are the terminal consequences of clinical sepsis. There is broad agreement amongst studies that lipopolysaccharide (LPS) does not directly activate PAR2; however, PAR2 can act as a powerful context-dependent modulator of LPS/Toll-like receptor 4 (TLR4) signalling, capable of amplifying, shaping or, in some settings, dampening LPS-driven inflammatory responses [49]. PAR2 does not transactivate TLR4; instead, the signalling pathways of these receptors converge at NF-κB. Consensus amongst studies is that systemic metabolic responses to (LPS)-induced endotoxaemia in PAR2^−/−^ do not differ from those of WT mice [50,51,52]. However, studies have shown LPS-induced PAR2 expression in tissues and affected PAR2 endothelial cell activities. Thus, PAR2 may be a bystander to regional or tissue-specific pathologies in sepsis. Indeed, researchers have proposed that PAR2 interactions with PAR1 may affect sepsis because they found that PAR2^−/−^ mice treated with a thrombin inhibitor decreased proinflammatory cytokine IL-6 expression and reduced mortality [51]. However, disparate observations have been made using double PAR2/PAR1 knockout mice (PAR2^−/−^/factor 2 (thrombin) receptor gene deficient (*f2r*^−/−^)), which did not affect survival from LPS endotoxaemia [52]. Further to support this notion of a baseline protective phenotype, PAR2^−/−^ mice did show lower lung content of myeloperoxidase under baseline conditions [50] prior to LPS, which subsequent studies demonstrated contributed to lung protection. On balance, the studies with PAR2^−/−^ have not led to PAR2 as a validated anti-sepsis or anti-endotoxaemia target.

PAR2^−/−^ have been used to study cardiac damage and remodelling in animal models of myocardial infarction and heart failure. After permanent ligation of the left anterior descending coronary artery (LAD), cardiac damage and dysfunction were reduced in PAR2^−/−^ compared to WT controls [8,9,53]. Similarly, PAR2^−/−^ reduced cardiac injury and prevented post-infarction remodelling [10]. PAR2^−/−^ mice showed smaller infarct size, less oxidative stress, lower mitogen-activated protein kinases (MAPK) phosphorylation, and pro-inflammatory gene expression compared to WT controls [10], consistent with a reduction in acute inflammatory signalling when PAR2 is absent. The same study reported that PAR2 deficiency was associated with reduced post-infarction cardiac remodelling and cardiac dysfunction. These findings suggest that PAR2 signalling may contribute to adverse cardiac responses following ischemia and support further investigation of PAR2-regulated pathways in ischaemic heart disease.

## 3. Discussion

### 3.1. Age-Dependent Cardiovascular Phenotype of PAR2 Deficiency

Ageing influences the cardiovascular phenotype of PAR2 deficiency and may affect PAR2 function. Young PAR2^−/−^ mice have a normal cardiac phenotype, while aged (1-year-old) PAR2^−/−^ mice develop cardiac fibrosis and diastolic dysfunction [4]. Recent pressure–volume studies demonstrated that PAR2^−/−^ mice exhibit elevated ventricular pressures, impaired relaxation kinetics, and increased mechanical work despite preserved cardiac output, indicating a pressure-dominant cardiac phenotype before overt systolic dysfunction develops [54]. These observations complement reports of age-dependent fibrosis and diastolic dysfunction in older PAR2^−/−^ mice [4]. The contrasting phenotypes observed in young and aged mice suggest that PAR2 may have a limited role in baseline cardiac function under normal conditions but becomes more important during age-related cardiovascular remodelling. A possible mechanism for this age-related change involves suppressed caveolin-1 regulation leading to increased cardiac PAR1 expression and profibrotic PAR1/TGF-β signalling [4]. PAR2 deficiency in aged models may remove normal restraint on collagen production, promoting myocardial fibrosis and ventricular stiffness, resulting in diastolic dysfunction. However, whether caveolin-1 dysregulation and increased profibrotic signalling directly cause diastolic dysfunction in aged PAR2^−/−^ remains unclear. In models of metabolic disease, PAR2-mediated vasodilation is preserved in younger animals but is reduced with age and a prolonged disease state [48]. This finding demonstrates how ageing impacts PAR2 function in metabolic disease models and how PAR2 deficiency may exacerbate cardiovascular dysfunction associated with ageing and metabolic diseases. Future studies should evaluate PAR2 function in aged models, as exclusive reliance on young animals may underestimate the cardiovascular consequences of PAR2 deficiency. These observations highlight ageing as an important determinant of PAR2 function and regulation in the cardiovascular system.

### 3.2. Intracellular and Cell-Specific PAR2 Signalling Pathways

PAR2 activates multiple intracellular signalling pathways that may contribute to the diverse cardiovascular phenotypes observed across experimental models. Following activation, PAR2 can signal through Gαq/11-dependent and β-arrestin-mediated pathways, including MAPK, ERK1/2, PI3K/Akt, and NF-κB signalling cascades. The relative contribution of these pathways varies by cell type, vascular bed, and disease state, helping explain the diverse roles of PAR2 across physiological and pathological settings [27,34,55].

Endothelial PAR2 regulates vascular function through multiple mechanisms, including nitric oxide-, endothelium-derived hyperpolarisation-, and prostanoid-dependent pathways [32,37,56,57,58]. In larger conductance arteries, PAR2-mediated vasodilation occurs primarily through NO signalling, whereas EDH contributes more prominently in smaller resistance arteries [32,56,59]. PAR2 activation can also induce MAPK- and NF-κB-dependent cyclooxygenase-2 (COX-2) expression and prostacyclin synthesis in endothelial cells [60,61,62], providing an additional mechanism through which PAR2 may influence vascular tone and endothelial function.

The functional consequences of endothelial PAR2 signalling appear to depend on the physiological setting. PAR2-mediated vasodilator responses are preserved in several models of hypertension and diabetes despite impaired responses to other endothelial agonists, suggesting that endothelial PAR2 signalling may remain functionally important under conditions associated with endothelial dysfunction [44,63,64,65,66]. Conversely, under inflammatory or metabolic disease conditions, PAR2 signalling has been proposed to alter prostanoid balance and may contribute to endothelial dysfunction through inflammatory signalling pathways [62,67]. These observations indicate that endothelial PAR2 may support vascular homeostasis in some settings while contributing to dysfunction in others.

In addition to endothelial signalling, PAR2 expressed in vascular smooth muscle cells (VSMCs) may influence vascular inflammation and remodelling. In cultured human VSMCs, PAR2 activation has been associated with increased COX-2 expression and activation of MAPK-, NF-κB-, and VEGFR-dependent signalling pathways [68,69]. These responses have been linked to pro-inflammatory and pro-atherogenic processes that may promote vascular remodelling and disease progression.

The diversity of signalling pathways activated by PAR2 in endothelial cells and VSMCs may help explain why global PAR2-deficient models exhibit heterogeneous cardiovascular phenotypes. Depending on the vascular bed and disease context, endothelial PAR2 may primarily influence vascular tone and endothelial homeostasis, whereas VSMC PAR2 may participate more directly in inflammatory and remodelling responses. These cell-specific signalling differences reinforce the need for endothelial-, VSMC-, and fibroblast-specific approaches to define the cardiovascular functions of PAR2 and interpret phenotypes observed in global PAR2-deficient models.

### 3.3. PAR2-PAR1 Cross-Talk

In addition to signalling diversity, interactions between PAR1 and PAR2 may further contribute to the disease- and tissue-specific effects of PAR2. Global PAR2^−/−^ mice exhibit enhanced responses to PAR1 activation, suggesting compensatory signalling and functional cross-talk between these receptors [23]. Mechanistically, PAR1 and PAR2 engage both overlapping and distinct downstream signalling pathways, which may permit reciprocal modulation of receptor activity [70]. PAR2 deficiency has also been associated with increased PAR1 expression, enhanced TGF-β-dependent collagen production, and cardiac fibrosis in aged mice [4]. These observations suggest that loss of PAR2 may alter PAR1-dependent signalling pathways involved in fibrosis and adverse cardiac remodelling. Although the mechanisms underlying PAR1–PAR2 interactions remain incompletely defined, available evidence indicates that altered PAR1 activity may underlie some phenotypes observed in global PAR2-deficient models.

### 3.4. Fibrosis Signalling Pathways

PAR2 deficiency may be associated with cardiac and vascular fibrosis, although the underlying signalling pathways are undefined. In the hearts of aged PAR2^−/−^ mice, increased PAR1 activity was associated with TGF-β-dependent collagen production, providing a possible pathway connecting PAR2 deficiency with cardiac fibrosis and impaired diastolic relaxation [4]. Similarly, PAR2 deficiency was associated with enhanced cardiac fibrosis in a model of angiotensin II-induced hypertension [6]. Upregulation of TGF-β and profibrotic gene expression was proposed as a possible mechanism, although the pathway responsible for the fibrosis was not established [6]. These observations suggest a potential antifibrotic role for PAR2 through TGF-β signalling. PAR2^−/−^ mice exhibited increased circulating ET-1 levels and enhanced aortic contraction to ET-1, which may indicate a profibrotic environment [5]. Findings from tight-skin mice indicated PAR2 expression may be a compensatory vasodilatory mechanism that opposes ET-1-related vascular dysfunction [5]. While these findings could suggest PAR2 reduces ET-1-induced endothelial dysfunction, a PAR2 mechanism that directly relates to fibrosis has not been characterised. In atherosclerosis, PAR2 may be pro-inflammatory and contribute to plaque instability, highlighting that PAR2 could act as a regulator of the balance between inflammatory and fibrosis pathways through different mechanisms [12,13,14]. PAR2 may regulate fibrosis through pathways that vary with disease setting and tissue type, although the molecular basis of these effects remains incompletely defined. Clarification of these pathways will improve interpretation of fibrosis-related phenotypes in PAR2-deficient models and may help guide future translational studies.

### 3.5. Inflammation Signalling Pathways

PAR2 participates in inflammatory signalling through multiple pathways that may influence cardiovascular disease progression in distinct pathological settings [55,71]. Experimental studies indicate that PAR2 can engage MAPK- and NF-κB-dependent signalling pathways [60,61], leading to expression of inflammatory mediators involved in immune cell recruitment, vascular dysfunction, and tissue remodelling. NF-κB represents a common downstream signalling node through which PAR2 may influence inflammatory responses in several cardiovascular disease settings.

In atherosclerosis models, PAR2 deficiency reduced lesion formation, macrophage accumulation, and expression of pro-inflammatory mediators, findings that were associated with reduced NF-κB signalling activity [13,14]. These observations suggest that PAR2 may contribute to vascular inflammation by promoting inflammatory gene expression and immune cell recruitment. Similarly, PAR2 deficiency reduced cardiac injury and adverse remodelling following myocardial infarction, which was associated with decreased MAPK phosphorylation and lower expression of pro-inflammatory genes [9].

**Figure 2:**
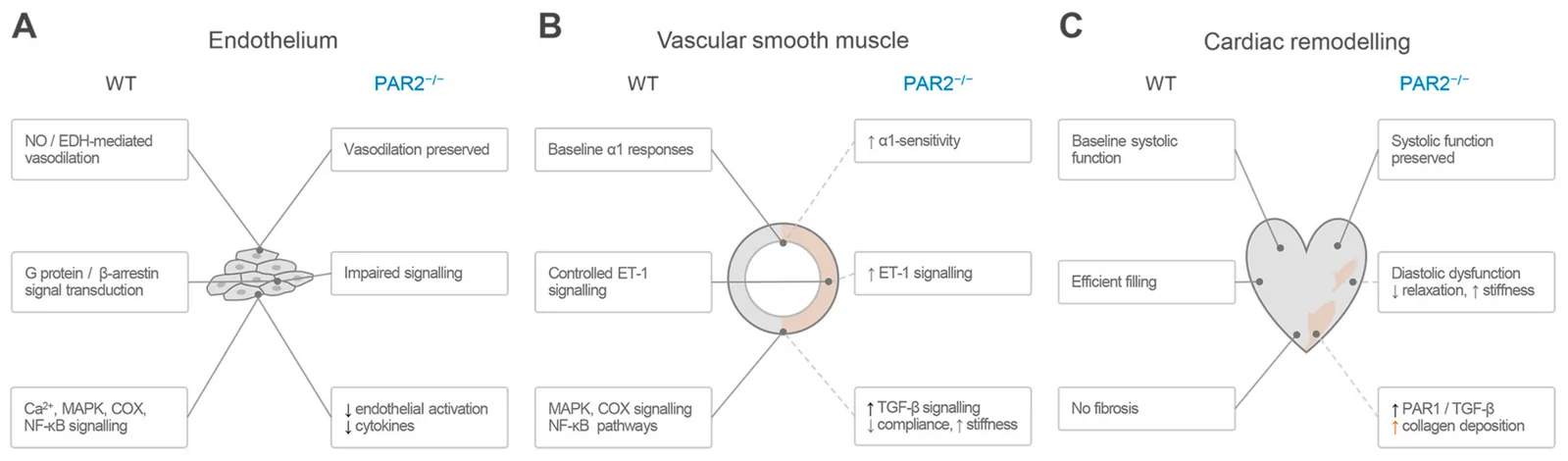
Proposed signalling mechanisms that may contribute to the cardiovascular phenotypes associated with PAR2 deficiency. (**A**) In endothelial cells, PAR2 signalling has been linked to regulation of vasodilation and inflammatory responses through nitric oxide-, endothelium-derived hyperpolarisation-, prostanoid-, MAPK-, and NF-κB-dependent pathways. (**B**) In vascular smooth muscle cells (VSMCs), loss of PAR2 may alter vasoconstrictor sensitivity and signalling pathways involved in vascular remodelling, inflammation, and extracellular matrix regulation. (**C**) In the heart, PAR2 deficiency has been associated with impaired diastolic relaxation, fibrosis, and altered PAR1- and TGF-β-related signalling. Together, the proposed pathways illustrate how cell-specific signalling, receptor cross-talk, and inflammatory and fibrotic responses may contribute to the context-dependent cardiovascular phenotypes observed in PAR2-deficient mice. This figure is an original conceptual summary created by the authors to synthesize the literature reviewed in this article. Solid lines indicate experimentally supported relationships reported in the literature, whereas dashed lines indicate proposed or associative pathways for which direct mechanistic evidence remains incomplete. Dashed pathways should therefore be interpreted as hypotheses generated from available evidence rather than established causal mechanisms. Abbreviations: α1-adrenoceptor; Ca^2+^, intracellular calcium; COX, cyclooxygenases-1/2; EDH, endothelium-derived hyperpolarisation; ET-1, endothelin-1; MAPK, mitogen-activated protein kinase; NO, nitric oxide; NFκB, nuclear factor kappa light chain-enhancer of activated B-cells; PAR1, proteinase-activated receptor 1; PAR2, proteinase-activated receptor 2; TGF-β, transforming growth factor-β.

PAR2 may also modify inflammatory responses through interactions with other signalling pathways. During lipopolysaccharide-induced inflammation, PAR2 and Toll-like receptor 4 (TLR4) signalling converge at NF-κB, allowing PAR2 to influence inflammatory responses without directly activating TLR4 [49]. Consistent with this concept, endothelial studies indicate that PAR2 activation can stimulate MAPK- and NF-κB-dependent COX-2 expression and downstream inflammatory mediator production [60,61,62]. These findings suggest that PAR2 may influence cardiovascular inflammation through interactions among NF-κB, MAPK, cyclooxygenase, and innate immune signalling pathways. The interplay between inflammatory and fibrotic responses may contribute to cardiovascular remodelling in experimental models and provides a framework for interpreting emerging observations in human disease. Figure 2 summarises cell-specific signalling pathways, PAR1–PAR2 interactions, and inflammatory and fibrotic mechanisms that may contribute to the cardiovascular phenotypes associated with PAR2 deficiency.

### 3.6. Human Relevance and Translational Implications

Clinical and translational studies indicate that PAR2 participates in vascular regulation, endothelial activation, inflammatory signalling, and processes relevant to cardiac remodelling. PAR2 activation produces direct vasomotor effects in vivo and contributes to regulation of vascular tone, supporting a physiological role for PAR2 signalling in the human circulation. Available evidence also indicates that PAR2 can exert both protective and detrimental effects depending on the disease context.

Consistent with experimental findings, PAR2 contributes to vasodilation, regulation of blood flow, endothelial function, and both NO-dependent and NO-independent vascular responses [37,38,39]. Conversely, activation of PAR2 in human endothelial cells can increase inflammatory mediator production, endothelial activation, and leukocyte recruitment [40,41,42,43]. These observations support the concept that PAR2 contributes to both protective vascular regulation and pro-inflammatory signalling, consistent with findings from experimental models of cardiovascular disease.

Findings in patients with HFpEF suggest that altered PAR signalling may be associated with myocardial fibrosis and diastolic dysfunction [4], supporting the potential clinical relevance of pathways identified in experimental models. These observations are consistent with experimental data linking altered PAR1/PAR2 signalling to profibrotic remodelling and impaired diastolic function. While the specific contribution of PAR2 to human disease remains incompletely defined, available evidence suggests that PAR signalling may influence mechanisms involved in pathological remodelling and ventricular dysfunction. These findings support further investigation of PAR1/PAR2-regulated pathways in myocardial fibrosis and diastolic dysfunction.

**Figure 3:**
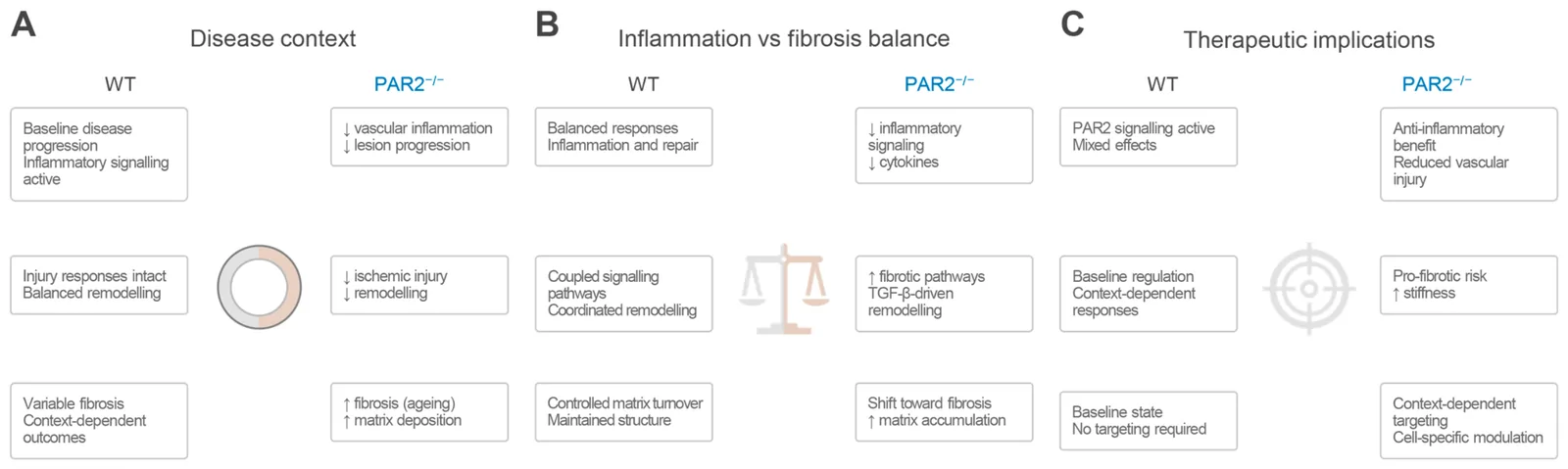
Context-dependent effects and therapeutic implications of PAR2 deficiency. (**A**) The impact of PAR2 deficiency varies across disease contexts, with reduced vascular inflammation and injury in atherosclerosis and acute ischemic models but increased fibrosis and matrix deposition in chronic and ageing conditions. (**B**) PAR2 may influence the balance between inflammatory and fibrotic responses, where reduced inflammatory signalling in PAR2^−/−^ is accompanied by a shift toward fibrosis and structural remodelling. (**C**) These context-dependent effects have therapeutic implications: PAR2 inhibition may provide anti-inflammatory benefit while increasing pro-fibrotic risk, highlighting the need for context-dependent and cell-specific targeting strategies. Figure 3 is an original conceptual summary created by the authors to illustrate the context-dependent cardiovascular effects and therapeutic implications discussed in this review. Abbreviations: PAR2, proteinase-activated receptor-2; TGF-β, Transforming growth factor-β.

Therapeutic targeting of PAR2 is complicated by its complex and sometimes opposing biological effects. Activation of PAR2 may improve vascular function, whereas systemic PAR2 inhibition has been associated with adverse cardiovascular and renal effects in some experimental settings [72]. PAR2 signalling bias adds further complexity, as biological responses may depend on the selectivity of PAR2 modulators for distinct downstream signalling pathways. Interactions between PAR1 and PAR2 signalling pathways may further influence disease outcomes by modifying downstream inflammatory and fibrotic responses [4,70]. In addition, cell-type-specific actions of PAR2 may produce distinct local and systemic biological responses. These complexities suggest that future therapeutic approaches may require tissue-specific, cell-specific, or signalling-selective modulation of PAR2 pathways to maximise beneficial cardiovascular effects while minimising unintended consequences. The findings reviewed here suggest that the cardiovascular consequences of PAR2 deficiency depend on tissue and disease context. Figure 3 summarizes disease-specific effects and potential therapeutic implications.

### 3.7. Experimental Models and Future Directions

Most evidence summarised in this review derives from global PAR2-deficient mice. Although these models have provided important insights into cardiovascular function, they cannot distinguish cell-specific effects and may be influenced by developmental compensation. Global deletion of PAR2 across all tissues makes it difficult to identify which cell populations contribute to the observed cardiovascular phenotypes. In addition, lifelong absence of PAR2 may permit adaptive changes in related signalling pathways during development, potentially modifying physiological and pathological responses. Cell-specific and inducible models may therefore provide a more precise understanding of the roles of PAR2 in different cardiovascular cell populations and help clarify the mechanisms underlying the diverse phenotypes observed in global PAR2-deficient mice.

Evidence from bone marrow transplantation studies further suggests that PAR2 expressed in different cell populations contributes distinctly to cardiovascular pathology. Transplantation of PAR2^−/−^ bone marrow reduced cardiac inflammation following myocardial infarction and attenuated vascular inflammation in atherosclerosis models, implicating haematopoietic PAR2 signalling in these responses [8,14]. Conversely, PAR2 expression on non-haematopoietic cells has been reported to support atherosclerosis through effects on macrophage infiltration [13]. These findings indicate that cardiovascular phenotypes observed in global PAR2-deficient mice likely reflect the combined contributions of multiple cell types. Accordingly, future studies should utilise tissue-specific, cell-specific, and inducible approaches to define the cardiovascular functions of PAR2 within distinct cellular compartments and identify therapeutically relevant PAR2-dependent pathways.

Endothelial cell-specific PAR2 deletion could clarify whether the vascular phenotypes observed in global PAR2-deficient mice arise primarily from loss of endothelial PAR2 signalling. PAR2 activation promotes endothelium-dependent vasodilation through nitric oxide-, hyperpolarisation-, and prostanoid-dependent pathways, while global PAR2^−/−^ mice exhibit modest elevations in arterial blood pressure [3,33,73]. Endothelial-specific models could therefore determine whether PAR2 directly regulates endothelial function, maintains vascular homeostasis, or protects against endothelial dysfunction in cardiovascular disease.

Vascular smooth muscle cell (VSMC)-specific PAR2 deletion could help distinguish smooth muscle-mediated effects from endothelial PAR2 signalling. Studies indicate that PAR2 activation promotes VSMC proliferation through intracellular signalling pathways linked to vascular growth and remodelling [74]. Such models could determine whether VSMC-expressed PAR2 contributes directly to vascular inflammation, remodelling, and structural adaptation independently of endothelial PAR2 signalling and clarify the role of VSMC PAR2 in cardiovascular disease.

Fibroblast-specific PAR2 deletion may help define the contribution of PAR2 to cardiac remodelling and fibrosis. Findings from global PAR2-deficient mice suggest that loss of PAR2 is associated with increased PAR1/TGF-β signalling, enhanced collagen production, and cardiac fibrosis [4]. Fibroblast-specific models could determine whether these profibrotic responses arise directly from fibroblast PAR2 signalling and clarify the contribution of fibroblast PAR2 to fibrosis and diastolic dysfunction.

Inducible gene deletion strategies, such as tamoxifen-inducible Cre-loxP systems, provide an additional approach for studying PAR2 function. When combined with cell-specific deletion, inducible models can help distinguish the direct consequences of PAR2 loss from adaptive developmental changes that may occur in constitutive knockout models. These approaches would also allow investigation of age-dependent PAR2 functions by comparing gene deletion before and after the development of age-related cardiovascular changes. Such studies may provide important insight into whether PAR2 contributes differently to cardiovascular physiology during ageing than during development or early adulthood.

Pharmacological modulation could complement genetic approaches by determining whether activating or inhibiting PAR2 reproduces cardiovascular phenotypes observed in knockout models. The selective PAR2 agonist 2fLIGRLO induces arterial vasodilation and may help define the vascular consequences of PAR2 activation [33,35]. Conversely, PAR2 antagonists such as AZ8838 and AZ3451 could be used to determine whether receptor inhibition prevents specific signalling pathways or cardiovascular dysfunction in selected disease contexts [75]. Pharmacological approaches may also help distinguish beneficial from detrimental PAR2 signalling pathways and determine whether signalling-biased modulation produces different outcomes than complete receptor activation or inhibition.

Although this review focuses primarily on cardiovascular phenotypes, PAR2^−/−^ mice have also been used to investigate regional and tissue-specific circulatory responses. For example, PAR2^−/−^ reduced ischemia-induced neovascularisation of the retina [22], and collateral formation in the hind limb [21], and more recently, PAR2^−/−^ prevented nitrovasodilator priming of migraine behaviours in a preclinical mouse model [76]. Furthermore, we recognize that metabolic [77,78,79] and embryonic [80] phenotype traits described in PAR2^−/−^ may influence cardiovascular outcomes, particularly where vascular disease risk or cardiac recovery from injury are concerned. These observations highlight the importance of considering regional and integrative physiological mechanisms when interpreting cardiovascular phenotypes associated with PAR2 deficiency.

The influence of biological sex on PAR2-dependent cardiovascular phenotypes remains poorly understood. Evidence regarding PAR2-specific interactions with biological sex and cardiovascular outcomes remains limited, as most published studies reviewed here, with a few exceptions [13,24,54], reported experiments in male PAR2^−/−^ mice only. The proliferation phenotype of primitive colonic epithelial cells exhibits sexual dimorphism in PAR2^−/−^ mice [81]. Given the interaction of sex hormones with cardiovascular health and disease in humans, future research should incorporate biological sex into experimental designs.

Overall, these approaches may help resolve the cellular origins and signalling mechanisms underlying the diverse cardiovascular phenotypes observed in PAR2-deficient mice. Improved understanding of these pathways will be important for interpreting the diverse cardiovascular functions of PAR2 and evaluating its potential as a therapeutic target.

## 4. Conclusions

PAR2 deficiency reveals a context-dependent role in cardiovascular regulation. Loss of PAR2 signalling preserves endothelial vasodilator function but shifts vascular responses toward increased vasoconstrictor sensitivity and structural remodelling.

Across experimental models, PAR2 deficiency has been associated with reduced inflammatory signalling and increased fibrosis in vascular and cardiac tissues, suggesting a divergence between inflammatory and matrix remodelling responses. While these observations occur with only modest changes in baseline hemodynamics, they may contribute to increased vascular stiffness and altered tissue structure in a context-dependent manner.

In the heart, PAR2 deficiency is associated with preserved systolic function but impaired diastolic relaxation with ageing, accompanied by increased fibrosis and reduced ventricular compliance.

Current evidence suggests that PAR2 may influence both inflammatory and fibrotic pathways in the circulatory system, although the mechanisms linking these responses remain incompletely defined. Future studies should clarify cell-specific functions and signalling pathways to improve understanding of the cardiovascular roles of PAR2 and inform development of targeted therapeutic strategies.

## Data Availability

No new data were created or analyzed in this study. Data sharing is not applicable to this article.

## Abbreviations

*apoE* ^−/−^	Apolipoprotein E gene knockout/deficient mouse
Ca^2+^	Intracellular calcium ion
COX-2	cyclooxygenase-2
CXCL1	C-X-C motif chemokine ligand 1
EDH	Endothelium-derived hyperpolarization
ET-1	Endothelin-1
ERK	Extracellular signal-regulated kinase
F2rl1	Factor 2 (thrombin) receptor-like 1 (PAR2) human gene
*f2rl1*	Factor 2 (thrombin) receptor-like 1 (PAR2) mouse gene
*f2rl1* ^−/−^	PAR2 gene knockout/deficient mouse
GPCR	G protein-coupled receptor
HFpEF	heart failure with preserved ejection fraction
IκBα	Inhibitor of nuclear factor-kappa B protein-alpha
IL-6	Interleukin-6
*ldlr* ^−/−^	Low density lipoprotein receptor gene knockout/deficient mouse
LPS	Lipopolysaccharide
MAPK	Mitogen-activated protein kinase
MMP-9	Matrix metalloproteinase-9
MCP-1	Monocyte chemoattractant protein-1
NF-κB	Nuclear factor-kappa B
NO	Nitric oxide
PAR2	Proteinase-activated receptor 2
PAR2-AP	PAR2-activating peptide
PI3K	Phosphoinositide 3-kinase
WT	wild-type genetic background (control) mice
PAR2^−/−^	PAR2-deficient/knockout animals, tissues or cells
TGF-β	Transforming growth factor-β
TNF-α	Tumour necrosis factor-α
TLR4	Toll-like receptor 4
VSMC	Vascular smooth muscle cell
